# Ryanodine Receptor Ca^2+^ Leak‐Induced Redistribution of Ca^2+^ in Dystrophic mdx Mouse Muscle

**DOI:** 10.1111/apha.70251

**Published:** 2026-05-14

**Authors:** Rhayanna B. Gaglianone, Cedric R. Lamboley, Taylor Dick, Aldo Meizoso‐Huesca, Daniel P. Singh, Bradley S. Launikonis

**Affiliations:** ^1^ School of Biomedical Sciences The University of Queensland Brisbane Queensland Australia

**Keywords:** Ca^2+^, confocal, muscular dystrophy, ryanodine receptor, skeletal muscle

## Abstract

**Aim:**

The dystrophic mdx mouse is a widely used model of Duchenne muscular dystrophy. Altered Ca^2+^ handling is a key feature, including increased Ca^2+^ leak through the ryanodine receptor (RyR1's), the primary Ca^2+^ release channel in skeletal muscle. Such leak has important downstream consequences for intracellular Ca^2+^ homeostasis. Here, we quantified basal compartmentalized Ca^2+^ levels in mdx muscle compared with wild‐type (WT).

**Methods:**

Single extensor digitorum longus muscle fibers from WT and mdx mice were mechanically skinned. Transverse tubule Ca^2+^ dynamics were assessed using confocal microscopy with fluorescent Ca^2+^ indicators during caffeine‐induced RyR1‐mediated Ca^2+^ release. Sarcoplasmic reticulum (SR) and mitochondrial Ca^2+^ contents were quantified using established depletion protocols combined with force measurements.

**Results:**

Consistent with previous reports, mdx fibers exhibited increased RyR1 Ca^2+^ leak. Absolute quantification revealed a reduction in SR Ca^2+^ content accompanied by a ~4‐fold increase in mitochondrial Ca^2+^ content. These shifts indicate a redistribution of intracellular Ca^2+^, triggered by the RyR1 Ca^2+^ leak to lower SR Ca^2+^ content and increase the Ca^2+^ permeability of the t‐system membrane, leading to an elevation in cytoplasmic and mitochondrial Ca^2+^ levels in mdx muscle.

**Conclusion:**

Redistribution of Ca^2+^ is a regulated process, proportional to RyR1 Ca^2+^ leak. In mdx muscle fibers, there is reduced SR and elevated mitochondrial and cytoplasmic Ca^2+^ compared to WT fibers. These alterations contribute to the dystrophic muscle pathology, likely through promotion of oxidative stress through increased reactive oxygen species production.

## 1 Introduction

Duchenne muscular dystrophy (DMD) is the most severe form of muscular dystrophy, with an incidence of 1:3500 male births. This disease occurs through mutations or other genetic rearrangements in the *DMD* gene leading to a complete absence of the protein dystrophin [1]. As a consequence of these genetic abnormalities, boys with DMD experience progressive muscle degeneration, muscle weakness, impaired muscle regeneration, and impaired ambulation [2]. Over time, continuous muscle damage progressively leads to the replacement of muscle fibers with fibrotic tissue and fatty acid deposits, which reduces muscle contractility and eventually results in cardiorespiratory failure at a relatively young age [3].

At a cellular level, the absence of dystrophin disrupts the dystrophin‐glycoprotein complex, losing the connection between the cytoskeleton, the contractile apparatus, and the extracellular matrix, reported by some to lead to membrane tears [1] or some other increase in Ca^2+^ permeability of the plasma membrane [2, 4, 5]. An important hallmark of DMD progression is the subsequent Ca^2+^ influx following membrane damage. The raised free cytosolic Ca^2+^ concentration ([Ca^2+^]_cyto_) stimulates the production of ROS through the activation of NADPH oxidase, which in turn modulates the functioning of surrounding proteins through post‐translational modifications (PTMs) [6, 7].

Another important change identified by [7] is the reduced stability of ryanodine receptor's (RyR1) closed state, causing this channel to leak more Ca^2+^. The increase in Ca^2+^ leak through the RyR1 triggers a cascade of events from the increase in Ca^2+^ permeability of the t‐system membrane to increasing the cytoplasmic and mitochondrial Ca^2+^ content [8, 9]. Such chronic increases in cytoplasmic and mitochondrial Ca^2+^ may underlie further generation of ROS and compound the progression of pathology.

The distribution of Ca^2+^ within the intracellular compartments of dystrophic mdx mouse muscle has not been previously characterized. Given the significance of the mdx mouse model in the study of DMD, we aimed to provide the first quantification of Ca^2+^ content within the sarcoplasmic reticulum (SR), mitochondria, and cytoplasm of mdx muscle. Our findings revealed an increased leakiness of the RyR1 and altered Ca^2+^ distribution in the muscle fibers of 14‐week‐old mdx mice compared to age‐matched wild‐type (WT) mice. 14‐week‐old C57Bl/6J mice WT and mdx were chosen because mdx mouse muscle is not yet affected by senescence and shows only mild inflammatory reaction and efficient muscular regeneration at this age. These data are expected to be valuable for advancing DMD research, especially where the mdx dystrophic mouse is used as the DMD model.

## 2 Material and Methods

### 2.1 Ethics and Animals

All experiments were conducted following approval by The University of Queensland Animal Ethics Committee. Male, 14‐week‐old C57Bl/6J mice WT and mdx were euthanized by cervical dislocation. The extensor longus digitorum (EDL) muscles were quickly excised and pinned onto a Sylgard‐coated Petri dish under a layer of paraffin oil. Single muscle fibers were isolated, and the sarcolemma was mechanically removed by microdissection along the fiber using a pair of jeweler's fine forceps [10].

### 2.2 T‐System Rhod‐5 N for Measuring Ca^2+^ Movements and Detecting RyR1 Ca^2+^ Leak

To assess the Ca^2+^ flux across the t‐system assay, rhod‐5 N salt was trapped within the sealed t‐tubules of the mechanically skinned fiber.

Briefly, a bundle of fibers was isolated and exposed to the dye solution containing 2.5 mM rhod‐5 N salt, 140 mM NaCl, 1 mM MgCl_2_, 5 mM glucose, 4 mM KCl, 10 mM HEPES, pH adjusted to 7.4. The bundles were incubated for 15 min at room temperature to permit the diffusion of the dye. Thereafter, single mechanically skinned fibers were isolated and mounted on a custom chamber containing a K^+^‐based solution, composed of 50 mM EGTA, 8 mM ATP, 10 mM creatine phosphate (CP), 90 mM HEPES, 36 mM Na^+^, 126 mM K^+^, 1 mM Mg^2+^, 0.05 mM and n‐benzyl‐p‐toluene sulphonamide (BTS). The cytoplasmic Ca^2+^ concentration ([Ca^2+^]_cyto_) was set to 85 nM. To fully deplete the Ca^2+^ stores from SR, the fibers were exposed to the K^+^‐based solution where caffeine was 30 mM, Ca^2+^ was nominally 0, and the [Mg^2+^] was lowered from 1 mM to 0.01 mM.

**FIGURE 1:**
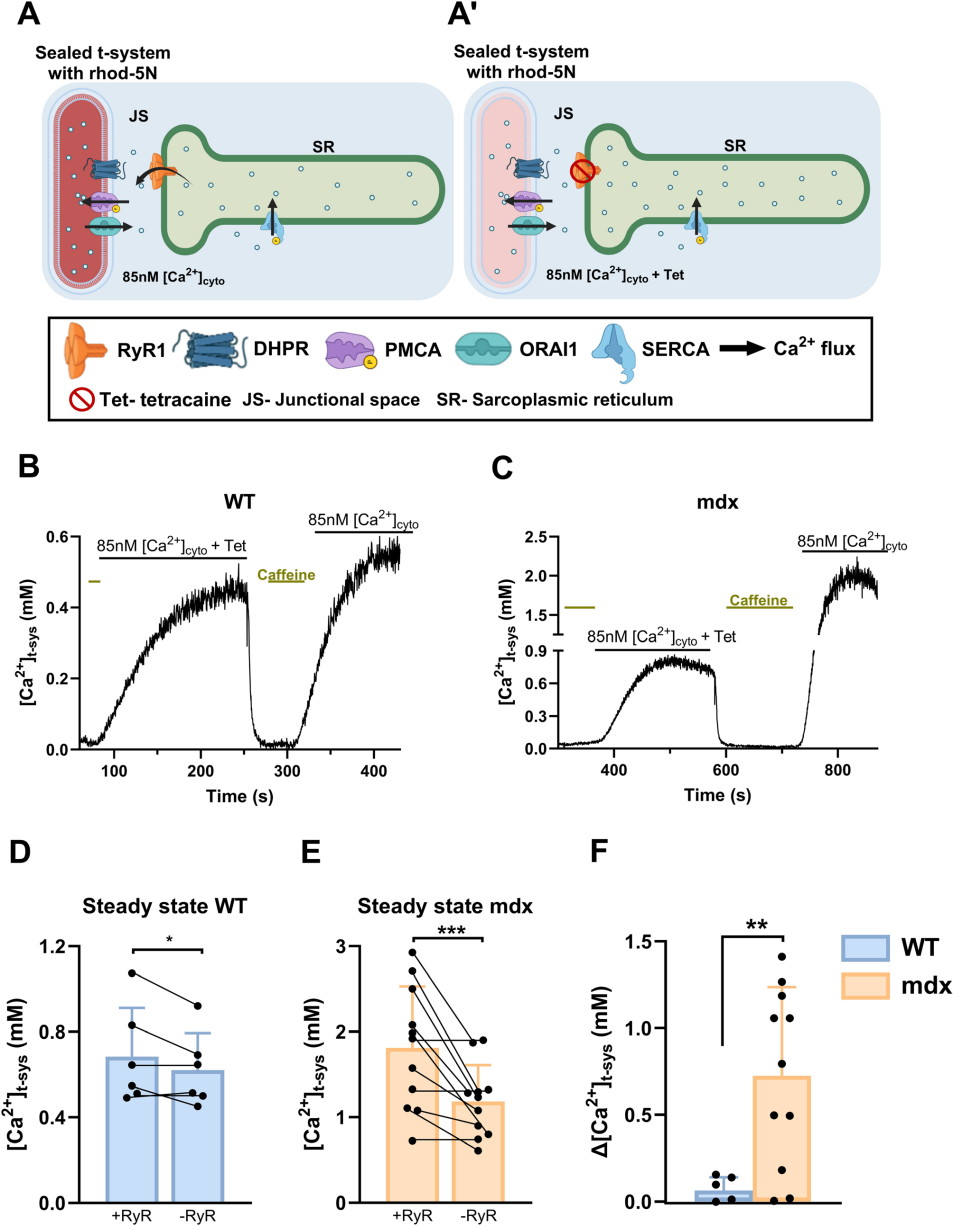
*T‐system Ca* ^2+^ *handling and RyR1 Ca* ^2+^ *leak in mdx muscle fibers*. (A) schematic diagram of rhod‐5 N trapped in the sealed t‐system as used to assess RyR1 Ca^2+^ leak. The functional RyR1 leaks Ca^2+^ into the junctional space (JS), increasing the local [Ca^2+^]_JS_ above that of the bulk cytoplasm, which is set by the internal bathing solution. The [Ca^2+^]_JS_ directly influences PMCA activity to pump Ca^2+^ into the sealed t‐system until an equilibrium is reached with t‐system Ca^2+^ leak to set the steady [Ca^2+^]_t‐sys_. The [Ca^2+^]_t‐sys_ is reported by the rhod‐5 N fluorescence signal. Introducing a RyR1 leak blocker, tetracaine (A'), causes a decrease in the [Ca^2+^]_JS_, and subsequently a decrease in the [Ca^2+^]_t‐sys_ to a new steady state as t‐system Ca^2+^ leak equilibrates with the adjusted Ca^2+^ pumping activity of the PMCA. (B) and (C) representative traces of [Ca^2+^]_t‐sys_ from WT and mdx respectively. The t‐system is fully depleted following the application of 30 mM caffeine. Substituting caffeine for a solution containing 85 nM Ca^2+^ in the presence of tetracaine (first transient) and then absence of tetracaine (second transient) allows the assessment of RyR1 Ca^2+^ leak to the [Ca^2+^]_t‐sys_ transient. (D) Summary of steady state [Ca^2+^]_t‐sys_ in WT and mdx fibers in the presence and absence of functional RyRs. In mdx, it is noticeable the difference between the first transient‐blocked RyR1 (− RyR1) and the second transient‐active RyR1 (+ RyR1). (E) Δ[Ca^2+^]_t‐sys_ provides the RyR1 Ca^2+^ leak index. Statistical test: Wilcoxon test, *p*‐values are: (D) *p = 0.03, (E) ***p = 0.001; and a Mann–Whitney test: (F) **p = 0.0067. Data is presented as mean ± SD. *n* WT = 3 mice, 6 fibers, *n* mdx = 3 mice, 11 fibers.

To obtain a record of t‐system Ca^2+^ flux, the cytoplasmic solution was exchanged between high caffeine and 85 nM Ca^2+^ solution. Initially, the administration of 30 mM caffeine in low Mg^2+^ and zero Ca^2+^ solution activates the RyR1 to fully deplete the SR Ca^2+^ stores. This depletion induces a unidirectional t‐system membrane Ca^2+^ flux via store‐operated Ca^2+^ entry (SOCE), causing a rapid decline of [Ca^2+^]_t‐sys_ to a low level (Figure 1B,C). The exchange from caffeine solution to a steady highly buffered solution at 85 nM Ca^2+^ terminates the SOCE and a rapid rise in the [Ca^2+^]_t‐sys_ (*t*) to a new steady state is achieved [9, 11].

After the [Ca^2+^]_SR_ depletion, the K^+^‐based solution containing 85 nM Ca^2+^ and 1 mM tetracaine was added to the fiber, increasing the [Ca^2+^]_t‐sys_ steady state to approximately 0.45 mM in WT and 0.80 mM in mdx. The administration of tetracaine (1 mM in K^+^‐based solution, dissolved in DMSO; control solutions had the same volume of DMSO) inhibits the RyR1 and so blocks the SR Ca^2+^ leak (Figure 1A'). Therefore, the transients obtained under tetracaine exposure are defined by [Ca^2+^]_cyto_ (85 nM) and are lower than the Ca^2+^ transients obtained with the RyR1 Ca^2+^ leak contribution.

The replacement of tetracaine solution with the caffeine‐containing solution depletes the SR reactivating the SOCE producing a new signal drop and the removal of tetracaine from the RyR1. The addition of 85 nM Ca^2+^ K^+^‐based solution without tetracaine causes an increase in the t‐system steady state at around 0.6 in WT and 1.9 mM in mdx. Because the RyR1 Ca^2+^ leak is active (Figure 1A), the [Ca^2+^] at the junctional space is higher than the 85 nM [Ca^2+^] established by the internal solution generating a new steady state that is higher than the previous one (Figure 1A–C). The difference obtained from the two steady states (+RyR1 and ‐RyR1) provides the RyR1 Ca^2+^ leak index [12].

Furthermore, the addition of F_max_ solution (5 mM Ca^2+^, 1 mM Mg^2+^, 4 mM K^+^, 140 mM Na^+^, 90 mM HEPES, 0.05 mM BTS, 0.05 mM Ionomycin) followed by F_min_ solution (50 mM EGTA, 8 mM ATP, 10 mM CP, 90 mM HEPES, 36 mM Na^+^, 126 mM K^+^) was used to determine saturation points of the t‐system rhod‐5 N fluorescence signal [12].

The equation used to calibrate t‐system rhod‐5 N and [Ca^2+^]_t‐sys_ was:

$${\left[{\mathrm{Ca}}^{2+}\right]}_{t-\mathrm{sys}}\left(t\right)=K_{D,\mathrm{Ca}}\frac{\left(F\left(t\right)-F_{\min}\right)}{\left(F_{\max}-F\left(t\right)\right)}$$

where the K_*D,Ca*_ is 0.872 mM, as previously established by Cully et al. [12]. F_*max*_ and F_*min*_ are the maximum and minimum fluorescence (respectively) of the dye within the fiber, and F(*t*) is the fluorescence intensity (arbitrary units) of the rhod‐5 N transient. Therefore, each individual skinned fiber had fluorescence converted to [Ca^2+^].

The greater variability of the determined [Ca^2+^]_t‐sys_ in the mdx than the WT fibers can be viewed in the Results. This is due to the greater number of mdx fibers presenting higher t‐system rhod‐5 N values than in the WT fibers. As rhod‐5 N nears saturation with Ca^2+^, the relationship between the dye signal and [Ca^2+^]_t‐sys_ becomes more exponential than linear. The result is a lowering of the resolution of the calibration and increasing the variability in the determination of [Ca^2+^]_t‐sys_ at higher levels of [Ca^2+^]_t‐sys_.

### 2.3 Confocal Settings

The fibers were imaged on the Olympus FV1000 confocal microscope equipped with a 0.9 numerical aperture (NA) 40× Plan‐Apochromat objective. Rhod‐5 N was excited with 543 nm HeNe laser, and the emission was filtered using a spectral detector. To track Ca^2+^ movements across the t‐system membrane, images were constantly recorded in *xyt* scanning with lasers positioned perpendicular to the fiber axis. For t‐system imaging, an aspect ratio of 512 × 128 pixels was used, and each frame of the *xyt* series was captured in 0.292 s. For analysis, a region‐of‐interest was drawn inside the boundaries of the fiber and fluorescence intensity was measured using ImageJ.

### 2.4 Measurement of Total Ca^2+^ in Single Mechanically Skinned Fibers

The intracellular Ca^2+^ content of a fiber was calculated based on the force produced by the fiber in response to membrane lysis, as established previously by Stephenson and others [9, 10].

First, the initial total amount of Ca^2+^ contained in a fiber was quantified by pre‐equilibrating the skinned fiber in a solution with a known concentration of BAPTA. BAPTA is a Ca^2+^ buffer derivative of EGTA that presents faster Ca^2+^ binding and release than EGTA. Therefore, for those experiments the administration of BAPTA is more appropriate. Later, the fibers are transferred to an emulsion of 10% Triton X‐100 and paraffin oil (TX‐oil) to lyse all membranous organelles and release any Ca^2+^ from within the fiber. Upon the membrane lysing, the Ca^2+^ released from the different intracellular organelles binds to the known amount of BAPTA present within the fiber and to troponin C (TnC) proteins. The pre‐equilibrating (BAPTA) was chosen such that the fiber produced a finite, non‐maximal force response upon lysis; this allowed for the calculation of the total amount of Ca^2+^ present in the fiber in absolute terms considering the BAPTA in the equilibration solution and the magnitude of the force response. Other skinned fiber preparations, prior to the TX‐oil lysing, were (i) fully depleted of their endogenous SR Ca^2+^ content by a 1 min exposure to a full release solution (0 Mg^2+^ + 30 mM caffeine), or (ii) fully depleted of their mitochondrial Ca^2+^ content by exposure to 25 μM FCCP. Due to the lysing of the membranes, fibers can only be used for a single measurement.

Because mitochondrial Ca^2+^ exchange occurs on a slower timescale than cytosolic Ca^2+^ buffering by BAPTA, the brief exposure used here provides a relative index of mitochondrial Ca^2+^ content that primarily reflects the pre‐existing cytosolic Ca^2+^ load. While some mitochondrial Ca^2+^ redistribution during BAPTA exposure cannot be excluded, such effects would be expected to occur similarly across experimental groups and therefore do not affect the comparative conclusions drawn.

### 2.5 Calculation of Ca^2+^ Release From Lysing Experiment

The total Ca^2+^ content within the fiber at the time of lysis ([Ca^2+^]T), expressed in millimoles per liter total fiber volume, was calculated as the sum of (i) the Ca^2+^ bound to BAPTA, (ii) the Ca^2+^ bound to all other high affinity binding sites in the fiber (predominantly Troponin C [TnC]). Briefly, the total amount of Ca^2+^ within a given fiber ([Ca^2+^]T) was calculated as follows:

1. The cytoplasmic free Ca^2+^ concentration ([Ca^2+^], in molar units) within the fiber at the peak of the force response elicited upon lysis was calculated from the relationship between force and [Ca^2+^] which was defined as being the Hill curve with values of pCa50 and Hill coefficient for that fiber of the specified mouse group.
2. The effective BAPTA within the fiber was taken as being 1.13 times the BAPTA of the pre‐equilibration solution, to account for the swelling of the fiber when initially placed in solution, and the fiber volume to which BAPTA was not accessible (i.e., that occupied by the SR, t‐tubular system, and mitochondria etc.)
3. The percentage of BAPTA with bound Ca^2+^ (%CaBAPTA) was determined from the size of the force response, the relevant force‐[Ca^2+^] relationship and the known Ca^2+^‐binding properties of BAPTA [13].
4. Ca^2+^ binds to the ATP and HDTA present, and the total of these and the free [Ca^2+^] was estimated as being ~9.6 × [Ca^2+^] [9, 14].
5. Finally, 0.015 mmol l‐1 was deducted from the total to consider the contaminating Ca^2+^ present in the BAPTA pre‐equilibration solution. The total Ca^2+^ content within the fiber at the time of lysis (expressed in millimoles per liter fiber volume) was thus calculated as follows:

$$\begin{array}{cc} \left[{\mathrm{Ca}}^{2+}\right]T= & \;1.13\times \left[\text{BAPTA}\right]\times \%\text{CaBAPTA}/100+\text{CaTnC}+9.6\times \left[{\mathrm{Ca}}^{2+}\right]\\ & \;\times 1000-0.015 \end{array}$$

To calculate the SR Ca^2+^ content of the fibers, the difference between the endogenous Ca^2+^ content and the non‐caffeine releasable Ca^2+^ content was determined. Specifically, each value of endogenous Ca^2+^ content was subtracted from the average non‐caffeine releasable Ca^2+^ content. This approach maintains the variability between individual fibers by using each value of endogenous Ca^2+^ content while using the average of the non‐caffeine releasable Ca^2+^ content that has been calculated from other preparations.

Likewise, to calculate the mitochondrial Ca^2+^ content, the difference between the non‐caffeine Ca^2+^ content and both the non‐caffeine releasable and non‐FCCP releasable Ca^2+^ content was determined. To establish the variability within groups, the individual fiber values for the non‐caffeine Ca^2+^ content were subtracted from the average of the non‐caffeine releasable and non‐FCCP releasable Ca^2+^ content from the same genotype.

### 2.6 Statistical Analysis

All statistical analyses were performed using GraphPad Prism 10.5. To determine statistical significance, Mann–Whitney and Wilcoxon tests were conducted. A *p*‐value of < 0.05 was considered statistically significant. All data are presented as mean ± standard deviation (SD).

## 3 Results

### 3.1 Detecting RyR1 Ca^2+^ Leak and t‐System Ca^2+^ Flux

RyR1 Ca^2+^ leak was determined by imaging [Ca^2+^]_t‐sys_ transients of skinned fibers (see Section 2 and Figure 1A [12]) from WT and mdx. The fibers were initially placed in an internal solution containing 85 nM Ca^2+^ and the continuous imaging of rhod‐5 N fluorescence signal from the t‐system was commenced on the confocal microscope. After establishing a baseline of ~30s, the internal bathing solution was exchanged for one containing 30 mM caffeine. This maneuver induced chronic opening of RyR1 causing depletion of [Ca^2+^]_SR_ stores, activating SOCE and, as a result, the [Ca^2+^]_t‐sys_ lowered. This can be observed as a signal drop on the traces of Figure 1B,C. Next, the 30 mM caffeine solution was exchanged for the internal solution containing 85 nM Ca^2+^, which terminates SOCE as the RyR1 shuts, allowing reloading of the SR and the t‐system with Ca^2+^.

By introducing the RyR1 inhibitor, tetracaine, under the same ionic conditions, the RyR1 leak is inhibited and the [Ca^2+^] in the junctional space ([Ca^2+^]_JS_) decreases (Figure 1A). Due to the decline of [Ca^2+^]_JS_, the activity of PMCA declines, resulting in a lowering of [Ca^2+^]_t‐sys_ to a new steady state. Figure 1B,C show representative traces obtained from WT and mdx, which demonstrate the differences in [Ca^2+^]_t‐sys_ transients induced by cytoplasmic solution exchange.

Figure 1D,E report the average steady state [Ca^2+^]_t‐sys_ in the presence of 85 nM [Ca^2+^] solution in both genotypes with active RyR1 (+RyR), and inhibited RyR1 (−RyR) in WT and mdx, respectively. The mdx presented higher steady state [Ca^2+^]_t‐sys_ (1.811 ± 0.717 mM) compared to WT (0.683 ± 0.228 mM) when RyR1 was active (+RyR1), indicating that the mdx displays increased RyR1 Ca^2+^ leak. The inhibition of RyR1 (−RyR1) with tetracaine decreased the steady state in both the WT and mdx fibers. One might expect that the steady state of mdx in the absence of RyR1 Ca^2+^ leak to be similar to WT values; however, after the RyR1 blockage, the mdx was still significantly higher (1.185 ± 0.424 mM) than the WT (0.621 ± 0.173 mM).

Figure 1D,E presents paired values of [Ca^2+^]_t‐sys_ (from the same fiber). In the mdx fibers, variability in RyR1 leakiness can be observed, which suggests heterogeneity in the leak response of the RyR1 at 14 weeks old. This heterogeneity could, in part, be because the t‐system rhod‐5 N in mdx fibers in the absence of tetracaine was approaching saturation with Ca^2+^, where the relationship between rhod‐5 N fluorescence and [Ca^2+^]_t‐sys_ becomes non‐linear and therefore more difficult to accurately calibrate than at lower levels of rhod‐5 N fluorescence [12].

In this scenario, where the RyR1 is blocked (−RyR1) with tetracaine, the [Ca^2+^]_JS_ in WT and mdx is established by the [Ca^2+^] of the internal solution, in this case 85 nM. Therefore, the [Ca^2+^]_t‐sys_ discrepancies under tetracaine exposure observed between the two genotypes rely on the balance of extrusion of Ca^2+^ by PMCA and the inward leak of Ca^2+^ through the t‐system membrane. In the presence of tetracaine, the raised t‐system steady state in mdx compared to WT fibers could be due to a combination of: (i) the elevated expression of PMCA detected on mdx muscle [15], (ii) Ca^2+^‐calmodulin binding to PMCA to increase its rate of Ca^2+^ extrusion and/or (iii) differences in t‐system Ca^2+^ channel densities such as Orai1 or transient receptor potential channels that Ca^2+^ can leak through between the genotypes [9, 16, 17, 18, 19].

To estimate the RyR1 Ca^2+^ leak index (Δ[Ca^2+^]_t‐sys_) (Figure 1F), we calculated the difference between steady state [Ca^2+^]_t‐sys_ with active RyR1 (+RyR1) and steady state [Ca^2+^]_t‐sys_ with inhibited RyR1 (−RyR1). Each data point was determined in the same fiber to improve the accuracy of this measurement. Consistent with other studies [7], the mdx presents greater Δ[Ca^2+^]_t‐sys_ (0.724 ± 0.523 mM) compared to WT (0.062 ± 0.077 mM).

### 3.2 Intracellular Ca^2+^ Distribution Across the Different Genotypes

Lamboley et al. [9] demonstrated that the spectrum of RyR1 Ca^2+^ leak causes a graded redistribution of intracellular Ca^2+^ among SR, cytosol, and mitochondria. The increased RyR1 leak suggests this should be the case in mdx muscle, so we decided to measure the organellar Ca^2+^ contents using the membrane‐lysis method [9, 10] (see Section 2).

**FIGURE 2:**
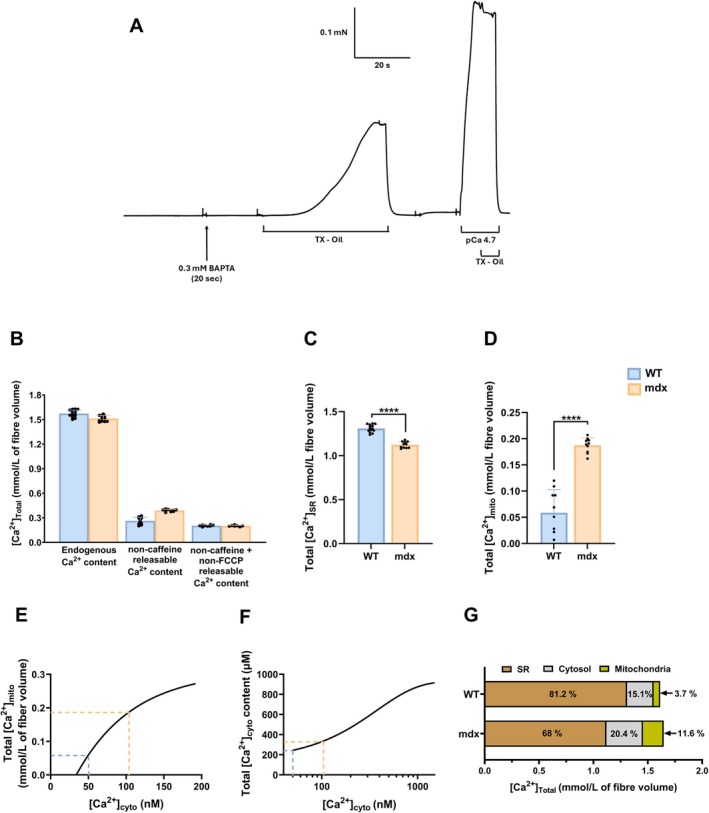
*Intracellular Ca* ^2+^ *redistribution in EDL fibers from WT and mdx mice*. (A) force recording from mdx muscle mechanically skinned fiber to measure fiber endogenous Ca^2+^ content. The fiber was first bathed in a 0.1 mM EGTA resting solution, then moved to 0.3 mM BAPTA, followed by membrane lysing in TX‐oil, which released all compartmentalized Ca^2+^ to generate a force response. TX‐oil was then washed out to relax the fiber. Finally, the fiber is equilibrated in a 50 mM EGTA relaxing solution prior to activation of maximal Ca^2+^‐activated force. (B) summary of endogenous (left), non‐caffeine releasable (middle) and non‐caffeine and on‐FCCP releasable Ca^2+^ content (right) in WT and mdx fibers. (C) total SR Ca^2+^ content and (D) total mitochondrial Ca^2+^ content in WT and mdx muscle fibers, as calculated from the data in B (see Methods). (E) Relationship between free [Ca^2+^]_cyto_ vs. total [Ca^2+^]_mito_ used to determine resting [Ca^2+^]_cyto_ in WT and mdx resting fibers. (F) Relationship between mitochondrial Ca^2+^ content vs. free [Ca^2+^]_cyto_ used to determine cytoplasmic Ca^2+^ content in WT and mdx fibers, see Lamboley et al. [9]. (G) SR, cytosol, and mitochondria total Ca^2+^ represented as a percentage of total fiber Ca^2+^ content in WT and mdx fibers. For (C) and (D), Mann–Whitney test revealed statistical differences in SR and mitochondrial Ca^2+^ content across the genotypes (***p < 0.0001). Data is presented as mean ± SD. *n* WT = 3 mice, *n* WT total SR content = 14 fibers, *n* WT total mitochondria content = 10 fibers. *n* mdx = 3 mice, *n* mdx total SR content = 11 fibers, *n* mdx total mitochondria content = 10 fibers.

An example force trace from an mdx fiber, pre‐equilibrated with BAPTA and sequentially exposed to the TX‐oil emulsion, wash‐out, and maximal Ca^2+^‐activation solution, is shown in Figure 2A. This protocol was used to estimate endogenous Ca^2+^ content. Corresponding values for WT and mdx fibers are presented in Figure 2B (left).

A pre‐exposure of the fiber to 30 mM caffeine to deplete the SR allows determination of the non‐caffeine releasable Ca^2+^ content of the fiber (Figure 2B, middle). The difference between the non‐caffeine releasable Ca^2+^ content and endogenous Ca^2+^ content is the SR Ca^2+^ content (Figure 2C). SR Ca^2+^ content (Figure 2C) was lower in mdx fibers (1.12 ± 0.037 mmol/L) compared with WT (1.31 ± 0.043 mmol/L; *p* < 0.0001).

Depletion SR Ca^2+^ is often associated with increased mitochondrial Ca^2+^ accumulation. Accordingly, a third maneuver of pre‐equilibration with caffeine and FCCP allowed estimation of mitochondrial Ca^2+^ content [9]. The non‐caffeine and non‐FCCP releasable Ca^2+^ content is presented in Figure 2B (right), and mitochondrial Ca^2+^ content was derived as the difference between non‐caffeine releasable and non‐caffeine/non‐FCCP releasable Ca^2+^ (Figure 2D).

Mitochondrial Ca^2+^ content was higher in mdx fibers (0.19 ± 0.014 mmol/L fiber volume) than in WT fibers (0.06 ± 0.044 mmol/L fiber volume; *p* < 0.0001), with all mdx mice exhibiting greater values than WT mice (Figure 2D). Elevated mitochondrial Ca^2+^ content is consistent with previously reported mitochondrial abnormalities in mdx muscle, including mitochondrial swelling and impaired oxidative phosphorylation [20, 21].

We have previously established a relationship between mitochondrial Ca^2+^ content and free [Ca^2+^]_cyto_ that allows free [Ca^2+^]_cyto_ and cytoplasmic Ca^2+^ content to be estimated [9, 22]. The estimation of free [Ca^2+^]_cyto_ is based on values of mitochondrial Ca^2+^ content measured by the membrane lysis technique used here [9, 23] and measures of resting [Ca^2+^]_cyto_ measured in intact fibers using Ca^2+^ electrodes [22]. Both these measurements were made with the same mouse genotypes. This relationship (Figure 2E) shows that free [Ca^2+^]_cyto_ in mdx is about 104 nM, compared to 50 nM in WT.

An important point here is that the SR pump is not influenced by the weakly Ca^2+^‐ buffered solution in which the fiber is initially bathed. All Ca^2+^ leaked from the SR is resequestered by the SR pump, as it outcompetes the low EGTA. Therefore, placing a fiber in 67 nM Ca^2+^ and low EGTA does not affect the SR Ca^2+^ content as the local [Ca^2+^] at the SERCA is determined by the RyR1 leak under these conditions [9, 23]. Furthermore, our preliminary experiments show that heavily Ca^2+^‐buffering the cytoplasm at low [Ca^2+^] (e.g., 67 nM as in Figure 2) during a brief exposure (2–3 min) does not affect the [Ca^2+^]_mito_ (0) or Ca^2+^ content. This is consistent with the measurement of different [Ca^2+^]_mito_ (0) values in the WT and mdx after the same brief exposure to 67 nM Ca^2+^ in the presence of 50 mM EGTA (Figure 2).

Next, the free [Ca^2+^]_cyto_ values allowed interpolation of cytoplasmic Ca^2+^ content from a previously established relationship model that takes into account all cytoplasmic Ca^2+^‐binding sites [9] (Figure 2F). Values of cytoplasmic Ca^2+^ content for mdx and WT were estimated to be about 335 μM and 244 μM, respectively.

Figure 2G shows a visual representation of intracellular Ca^2+^ content distribution in each genotype as a percentage of total Ca^2+^ content. In the dystrophic muscle fibers, 68% of the total intracellular Ca^2+^ content is in the SR, compared to 81.2% of WT. For this result to be achieved at steady state, RyR1 Ca^2+^ leak must have triggered a change in t‐system membrane Ca^2+^ permeability [9, 24] allowing the cytoplasmic Ca^2+^ content to remain raised, which in turn raises the mitochondrial Ca^2+^ content. This could be directly associated with increased oxidative stress, abnormal mitochondrial functioning, and loss of force production observed in dystrophic muscles [4, 7, 21, 25].

## 4 Discussion

**FIGURE 3:**
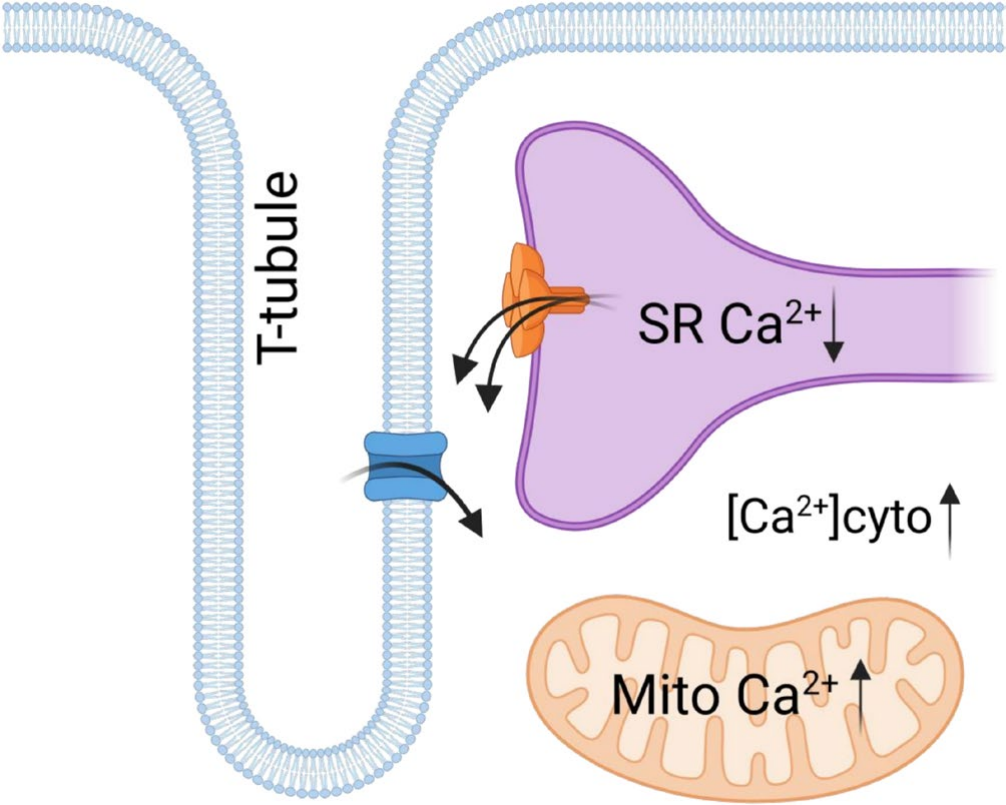
Redistribution of Ca^2+^ in dystrophic mdx mouse muscle. RyR1 Ca^2+^ leak is responsible for depleting SR Ca^2+^ content. This triggers a net influx of Ca^2+^ across the t‐system membrane to persistently raise cytoplasmic Ca^2+^ levels. Raised cytoplasmic Ca^2+^ levels drive Ca^2+^ into the mitochondria to raise the basal level of Ca^2+^. These alterations from the healthy (wild‐type) state may drive the generation of reactive oxygen species inside the mitochondria.

The dystrophic mdx mouse has been a heavily used model for studying DMD. In this study, we confirm that the mdx mouse muscle exhibits a leaky RyR1 at a relatively young age. Here, we present novel measurements of SR, mitochondrial, and cytoplasmic Ca^2+^ content in mdx muscle. The observed Ca^2+^ redistribution in the mdx mouse is consistent with how RyR1 Ca^2+^ leak was shown to drive this process in *RYR1* gain‐of‐function mutant mouse muscle [9]. That is, the RyR1 Ca^2+^ leak increases t‐system membrane Ca^2+^ permeability to maintain a raised resting [Ca^2+^]_cyto_. This is a compensatory maneuver by the fiber to maintain a similar total fiber Ca^2+^ content by increasing cytoplasmic Ca^2+^ when the SR Ca^2+^ content is lowered by chronic leak through RyR1 (Figure 3).

Redistributing fiber Ca^2+^ content has multiple advantages for the muscle with a leaky RyR1. There is a partial saturation of troponin C with Ca^2+^ in the resting fiber that reduces the load on Ca^2+^ release from SR during EC coupling to reach the same level of force compared to the muscle without a leaky RyR1. Additionally, the raised [Ca^2+^]_cyto_ also sets the steady state mitochondrial Ca^2+^ content, which is an important factor in the regulation of mitochondrial respiration [9, 23, 26, 27, 28].

However, in cases of chronically raised or increasing RyR1 leak over long periods, there can be a threshold that is passed where cytoplasmic and mitochondrial Ca^2+^ reach levels that generate levels of ROS that oxidize Ca^2+^‐handling proteins. This scenario perpetuates the dystrophic state, as recently shown in a mouse model of limb‐girdle muscular dystrophy 2B/R2 (LGMD [29]). Oxidation of plasma membrane Ca^2+^‐handling proteins slows Ca^2+^ extrusion to maintain the persistently raised cytoplasmic Ca^2+^, correlating with the development of fiber fibrosis.

While oxidative phosphorylation dysfunction has been identified at a similar age of mdx mouse as we have used here [30], we do not expect that maintaining steady‐state cytoplasmic ATP to be compromised at rest. First, it should be noted that the ATP hydrolysis rate at the SR Ca^2+^ pump required to maintain SR Ca^2+^ content in the resting muscle is 2‐orders of magnitude below the maximal rate of the pump. Therefore, the relative demand on ATP resynthesis is low [31, 32]. Furthermore, compared to mice with *RYR1* gain‐of‐function mutation or a LGMD model mouse, steady‐state SR and mitochondrial Ca^2+^ content in mdx fibers in the presence of RyR1 Ca^2+^ leak are maintained within expected levels [9, 29]. Regarding the increase in mitochondrial Ca^2+^ content of mdx fibers, it is the Ca^2+^ level in the cytoplasm and mitochondrial membrane potential that drive Ca^2+^ into the mitochondria to set its resting Ca^2+^ content in a balance with extrusion mechanisms [23, 26].

In the mdx mouse, there have been claims of microtears, transient receptor potential channels and SOCE inducing an increase in the resting [Ca^2+^]_cyto_ [2, 5, 33, 34, 35, 36]. Our results suggest that the resting [Ca^2+^]_cyto_ is raised in a controlled fashion, in response to the increased leak of Ca^2+^ through the RyR1. Furthermore, the increased [Ca^2+^]_mito_ is also an expected consequence of the increase in [Ca^2+^]_cyto_ and does not indicate any dysfunction in mitochondrial Ca^2+^ handling.

The redistribution of Ca^2+^ in the mdx fiber is a likely trigger for ROS generation, as suggested by earlier studies [7]. This is consistent with reports showing that treatment with antioxidants in mdx muscle alleviates some aspects of dystrophinopathy [25]. However, sustained raised [Ca^2+^]_cyto_ underlies the pathophysiological dysfunction in dystrophic muscles caused by other means than ROS. Elevated levels of [Ca^2+^]_cyto_ have been associated with increased activity of Ca^2+^‐dependent proteases, such as calpains, which result in proteolytic processing of several myofibrillar proteins like myosin, troponin, and tropomyosin, leading to loss of force [20, 37]. In accordance with this notion, inducing the overexpression of calpastatin, the only endogenous calpain inhibitor identified, reduces necrosis and improves muscle regeneration [38].

Moreover, in line with this rationale, therapeutic strategies aiming to reduce the [Ca^2+^]_cyto_ either pharmacologically or increasing the expression of key proteins have shown promise in mitigating the muscular dystrophy. Dystrophic muscles treated with streptomycin or taurine exposure, or those with increased expression of SERCA, evidenced reduced myonecrosis, fibrosis, inflammation and calpain activity, alongside improved Ca^2+^ cycling during EC coupling, force generation and mitochondrial respiration [39, 40, 41, 42, 43]. Altogether, these findings indicate that chronic elevation of [Ca^2+^]_cyto_ affects critical muscle functions in DMD, such as muscle contraction and relaxation, and that alterations in resting [Ca^2+^]_cyto_ levels are a major contributor to DMD pathophysiology [44].

Our results provide absolute values for compartmentalized Ca^2+^ levels in mdx muscle and demonstrate how these levels have shifted from those in the WT muscle. These findings show that in 14‐week‐old mdx mouse muscle the Ca^2+^ signaling pathway initiated by RyR1 Ca^2+^ leak remains functionally intact in the mdx mouse muscle, providing a valuable framework for evaluating the efficacy of therapeutic interventions. Looking forward, future studies coupling the redistribution of Ca^2+^ in mdx fibers with oxidative stress or mitochondrial respiration and exploring these issues also in slow‐twitch fibers will advance the field further.

## Author Contributions

**Bradley S. Launikonis:** conceptualization, funding acquisition, writing – review and editing, writing – original draft. **Daniel P. Singh:** writing – review and editing, formal analysis, data curation. **Taylor Dick:** formal analysis. **Rhayanna B. Gaglianone:** conceptualization, writing – original draft, data curation, writing – review and editing. **Cedric R. Lamboley:** data curation. **Aldo Meizoso‐Huesca:** data curation, writing – review and editing.

## Funding

This work was supported by an Australian Research Council Discovery Project (DP200100435) and a National Institute of Health (USA) R01 (AR082533) subcontract to BSL.

## Conflicts of Interest

The authors declare no conflicts of interest.

## Data Availability

The data that support the findings of this study are available from the corresponding author upon reasonable request.
